# Subcellular Trafficking of Mammalian Lysosomal Proteins: An Extended View

**DOI:** 10.3390/ijms18010047

**Published:** 2016-12-28

**Authors:** Catherine Staudt, Emeline Puissant, Marielle Boonen

**Affiliations:** Physiological Chemistry Laboratory-URPhyM, Narilis, University of Namur, 61 rue de Bruxelles, 5000 Namur, Belgium; catherine.staudt@unamur.be (C.S.); emeline.puissant@unamur.be (E.P.)

**Keywords:** lysosome, trafficking, unconventional, mannose 6-phosphate, alternative receptor, sorting motif

## Abstract

Lysosomes clear macromolecules, maintain nutrient and cholesterol homeostasis, participate in tissue repair, and in many other cellular functions. To assume these tasks, lysosomes rely on their large arsenal of acid hydrolases, transmembrane proteins and membrane-associated proteins. It is therefore imperative that, post-synthesis, these proteins are specifically recognized as lysosomal components and are correctly sorted to this organelle through the endosomes. Lysosomal transmembrane proteins contain consensus motifs in their cytosolic regions (tyrosine- or dileucine-based) that serve as sorting signals to the endosomes, whereas most lysosomal acid hydrolases acquire mannose 6-phosphate (Man-6-P) moieties that mediate binding to two membrane receptors with endosomal sorting motifs in their cytosolic tails. These tyrosine- and dileucine-based motifs are tickets for boarding in clathrin-coated carriers that transport their cargo from the trans-Golgi network and plasma membrane to the endosomes. However, increasing evidence points to additional mechanisms participating in the biogenesis of lysosomes. In some cell types, for example, there are alternatives to the Man-6-P receptors for the transport of some acid hydrolases. In addition, several “non-consensus” sorting motifs have been identified, and atypical transport routes to endolysosomes have been brought to light. These “unconventional” or “less known” transport mechanisms are the focus of this review.

## 1. Introduction

In the 1950s, Christian de Duve and colleagues made the peculiar observation that, when rat liver is homogenized in isotonic sucrose and fractionated into subcellular fractions by centrifugation, freezing/thawing of these fractions is required to get an accurate measurement of the total activity of several hydrolases with acidic pH optimums. As this treatment induces membrane rupture, it was suggested that the latent enzymes are confined within “membrane sacs” and are thus inaccessible to the exogenous substrates used in these activity assays. The subsequent findings that these enzymes co-distribute in rat liver subcellular fractions, and that their distribution profile (i.e., total amount, and enrichment level over total proteins in each fraction) differs from those reported for proteins located in other cellular structures led to the discovery of lysosomes ([1], reviewed by Sabatini and Adesnik [2]). Today, proteomic analyses have revealed that the lumen of lysosomes contains approximately 60 different acid hydrolases, and that the lysosomal membrane is spanned by many transmembrane proteins [3,4,5,6,7]. These include structural proteins, a transmembrane vATPase complex that generates an intraluminal acidic environment in which acid hydrolases are active, as well as a large set of transporters that transfer the enzyme degradation products in the cytosol. In addition, the lysosomal proteome comprises of many membrane-associated proteins that are recruited from the cytosol.

The lysosome relies on this large arsenal of proteins to assume its main function, i.e., the break-down of macromolecules delivered by endocytosis or autophagy into primary components that can be recycled to the cytosol to re-enter anabolic reactions. When unable to degrade these macromolecules, or to translocate their degradation products to the cytosol, the abnormal accumulation of material in the lysosomes causes lysosomal and cellular dysfunctions. To date, approximately fifty lysosomal storage diseases have been reported, many of them characterized by neurodegeneration, severe organ failure, and premature death [8,9]. Lysosomal alterations have also been associated with the negative evolution of other pathologies, including cancer, atherosclerosis, and Alzheimer’s and Huntington’s disease. The study of the underlying causes of lysosomal dysfunctions has pointed out that to maintain a well-oiled lysosomal machine and hence prevent deleterious cellular/tissue alterations, the cells must express all required lysosomal proteins but, most importantly, they also need to efficiently and specifically target them to the lysosomal compartment. To meet this second requirement, the cells rely on several intracellular trafficking machineries that transport newly synthesized lysosomal membrane or soluble proteins to their residence site within the cells. The canonical endolysosomal sorting pathways are based on the specific recognition of consensus sorting motifs located in cytosolic regions of the endolysosomal transmembrane proteins, or of mannose 6-phosphate (Man-6-P) residues exposed on the oligosaccharidic chains of acid hydrolases, by vesicular transport machineries that transfer cargoes between cellular compartments (reviewed in [10,11,12,13,14,15]). However, an increasing number of observations indicate that some lysosomal proteins can reach lysosomes by “non-conventional” transport mechanisms. While some of them have been known from quite some time, recent advances in this field reveal that these mechanisms are much more numerous than could have been anticipated, and that they rely on a wide array of molecular bases. Our aim herein is to provide an updated overview of the different types of motifs and transport machineries that mediate the biosynthetic transport of mammalian lysosomal proteins and, as such, control lysosomal function. We will only provide a brief description of the classical lysosomal sorting mechanisms for comparison purposes, as these have already been extensively discussed by others.

## 2. Subcellular Trafficking of Lysosomal Transmembrane Proteins

### 2.1. Classical Sorting Pathways

Lysosomal transmembrane proteins are synthesized in the endoplasmic reticulum (ER). After passage through the Golgi apparatus, they are either sorted directly from the trans-Golgi network (TGN) to the endosomes and lysosomes or indirectly, i.e., they are first sorted to the cell surface from where they enter the endocytic pathway. Both direct and indirect routes rely on clathrin-coated vesicles to carry the proteins from the TGN or plasma membrane (PM) to the endosomes. Several coat proteins referred to as “clathrin-adaptor proteins” are involved in protein packaging in the clathrin-coated carriers. They recognize short amino acid motifs (i.e., tyrosine- or dileucine-based motifs) located in a cytosolic region of the lysosomal transmembrane protein, usually in its N- or C-terminal end, though not always [11,12,13,14,16,17]. Tyrosine-based sorting signals fit to the (Fx)NPXY and (G)YXXφ consensus sequences (where Phi is a bulky hydrophobic amino acid), and dileucine-based signals conform to the [D/E]XXXL[L/I] or DXXLL sequences. At the TGN, signals of the (G)YXXφ- and [D/E]XXXL[L/I]- types are recognized by the clathrin adaptor complex AP-1, whereas DXXLL motifs bind to monomeric clathrin adaptor proteins known as GGAs (Golgi-localizing, gamma-adaptin ear domain homology, ARF-binding proteins). At the PM, the adaptor protein complex AP-2 recognizes NPXY, YXXφ and [D/E]XXXL[L/I] signals. In addition, NPXY signals can also bind to other cell surface clathrin-associated proteins, including DAB2, Numb and ARH (Autosomal recessive hypercholesterolemia). Two additional adaptor complexes are found at the TGN and on endosomal membranes, AP-3 and AP-4, which exhibit sequence homologies with AP-1 and AP-2. However, by contrast to the first two members of this family, AP-3 and AP-4 appear to be involved in both clathrin-dependent and clathrin-independent vesicular transport mechanisms. Both the YXXφ and [D/E]XXXL[L/I] motifs are recognized by AP-3, whereas only YXXφ motifs seem to bind to AP-4. AP-5, the last member of this adaptor protein family is recruited on endolysosomes and does not associate with clathrin. Although its function remains unclear, it has been reported that enlarged endolysosomes accumulate in AP-5-deficient cells, suggesting that this adaptor may also be involved in endolysosomal protein trafficking [18].

### 2.2. Targeting of Lysosomal Transmembrane Proteins to the Lysosome by Non-Conventional Mechanisms

While many lysosomal transmembrane proteins and lysosomal acid hydrolase membrane transporters (see Section 3) use these tyrosine and dileucine motifs-dependent trafficking pathways via clathrin-coated vesicles to travel to the endosomes and lysosomes, a growing number of reports highlight alternatives to these conventional transport mechanisms. First, several motifs that do not conform to the canonical tyrosine and dileucine signals can also mediate the sorting of lysosomal transmembrane proteins to the lysosome. Next, the trafficking of some lysosomal membrane proteins depends on their post-translational modifications, such as *N*-glycosylation and covalent lipid attachment, or on a particular transmembrane domain. Finally, the association of some transmembrane proteins with other proteins can sometimes drive their subcellular trafficking. An overview of lysosomal transmembrane proteins that use these atypical or less conventional transport mechanisms to reach their residence site in the cell is provided in Table 1 and discussed hereafter.

#### 2.2.1. Atypical Sorting Motifs Identified in Lysosomal Transmembrane Proteins

An YFPQA motif has been identified as a lysosomal sorting signal in the 3rd cytosolic loop of cystinosin, a cystine transporter whose deficiency causes cystinosis, an autosomal recessive lysosomal storage disorder that mostly affects the kidneys and eyes. This atypical amino acid sequence and a classical (G)YXXφ motif located in the C-terminal tail of the protein (GYDQL) are both required for efficient sorting to the lysosomes [19,20]. While the YXXφ motif is recognized by the adaptor protein AP-3 [20], how the YFPQA sequence mediates lysosomal sorting has not yet been resolved.

Several other proteins rely, at least partly, on tyrosine or leucine residues that are not included in a classical YXXφ, D/EXXXL/LI or DXXLL setup for sorting to endolysosomes, including VAMP7 and LAPTM5. VAMP7 is a protein of the SNARE family (soluble NSF (*N*-ethylmaleimide-sensitive factor) attachment receptors) involved in the fusion of late endosomes/lysosomes. When in a cis-SNARE complex, the cytosolic longin domain of VAMP7 is recognized by AP-3 and by the endocytic adaptor Hrb and, while the key residues for binding to these proteins are L43 and Y45, neither of these amino acids belongs to a consensus sorting motif [21,22,23]. In the polytopic protein LAPTM5 (Lysosomal-associated transmembrane protein 5), L/PPXY (PY) motifs located in cytosolic portions mediate binding to the ubiquitin ligase Nedd4, which, in turn, promotes the recruitment of an ubiquitinated form of the TGN adaptor GGA3 on the ubiquitin-interacting motif of LAPTM5 (LKVALPSYEE, consensus residues are underlined), and LAPTM5 transport to lysosomes [24]. Similarly, Nedd4 participates in the transport of the two other members of the LAPTM family (LAPTM4a and LAPTM4b) to the lysosomes in a PY motif-dependent manner [25].

Another category of atypical endolysosomal sorting signals is referred to as “extended acidic dileucine signals”, which are found in TMEM106B (EXXXXXLI) and CLN3 (EEEX(8)LI) [26,27,28]. TMEM106B is a protein associated with frontotemporal lobar degeneration [29], and the CLN3 encoding gene, which is mutated in the neurodegenerative pathology referred to as Batten disease, is one of the genes with the highest carrier frequency in cases of neuronal ceroid lipofuscinoses in the USA [30,31]. The EXXXXXLI signal localizes to the cytosolic N-terminal region of TMEM106B, whereas in CLN3, its EEEX(8)LI signal is in the 2nd cytosolic loop and binds to AP-1 and AP-3 [26,32]. The C-terminal region of CLN3 also contains an M(X)9G sequence that contributes to its sorting to the lysosomes [27,28]. Moreover, it has been reported that the transport of CLN3 and TMEM106B to the lysosomes is modulated by post-translational modifications, which will be detailed in Section 2.2.2 and Section 2.2.3. Hence, it appears that, while atypical dileucine signals can contribute to the lysosomal sorting of some proteins, additional trafficking information may be required for their efficient sorting to the lysosomes. The transport of G-protein coupled receptor 143 (GPR143 or OA1 for ocular albinism type I protein) to melanosomes in pigmented cells, and to endolysosomes in pigmented and non-pigmented cells, is in accordance with this view. To significantly alter the trafficking to these sites (i.e., to redirect the protein to the PM), an unconventional dileucine signal located in a cytosolic loop (SLLKGRQGIY) must be mutated simultaneously with a tryptophan and glutamic acid (WE) motif located in the C-terminal tail [33].

However, not all atypical signals revolve around tyrosine or dileucine amino acids (e.g., the M(X)9G and WE motifs mentioned above). This point is further supported when looking at MLN64, a lysosomal protein involved in cholesterol transport, whose sorting depends on a KSASNP motif located in its C-terminal START domain [34]. This motif mediates binding to the cytosolic protein 14-3-3 independently of phosphorylation and, when this interaction is prevented by alanine substitutions of amino acids K to N, MLN64 accumulates at the PM and exhibits a delayed transport to late endosomes from this site. Isoforms of the 14-3-3 family are known to modulate the subcellular localization of several soluble and transmembrane proteins through binding to phosphoserine or phosphothreonine motifs. Regarding soluble proteins, it has been proposed that their binding to 14-3-3 hides or exposes sorting signals that control their subcellular localization [35], e.g., the lysosomal gene network regulator TFEB, which is retained in the cytoplasm when bound to 14-3-3 proteins, and translocates to the nucleus when released from this association [36]. How 14-3-3 proteins regulate the subcellular trafficking of transmembrane proteins is less well understood, as there are not as many examples reported in the literature. It is usually assumed that the 14-3-3 masks (or unmasks upon dissociation) trafficking signals, such as RXR motifs that retain transmembrane proteins in the ER when correctly exposed [37]. It has also been shown that the AP-2-mediated internalization of the Na^+^/K^+^-ATPase requires its binding to phosphoinositide 3-kinase and the activation of this kinase, which is dependent on the channel association with 14-3-3 [38]. Based on this finding, it is tempting to suggest that 14-3-3 modulates the recognition of MLN64 by adaptor or accessory proteins involved in its internalization from the PM [34]. However, the 14-3-3 binding site of MLN64 is located in the C-terminal region [34] while, according to Zhang et al., the N-terminal domain (which includes the transmembrane regions of the protein) contains the information for efficient internalization from the cell surface [39]. Further work will be needed to investigate how the C-terminal tail, and its association with 14-3-3, mediates the sorting of MLN64.

#### 2.2.2. Sorting Determinants Located in the Luminal Domain of Lysosomal Transmembrane Proteins

The lysosomal sorting of TMEM106B is not only mediated by the extended dileucine signal located in its N-terminal region (see above), but also depends on its 4th and 5th *N*-glycosylation sites [26,40]. Indeed, mutation of the 4th site prevents the anterograde transport of TMEM106B to the endolysosomes by causing its retention in the ER (endoplasmic reticulum). While this likely results from a folding defect, mutation of the 5th site induces relocalization of TMEM106B at the cell surface, suggesting that this modification controls its lysosomal sorting [40]. Perhaps TMEM106B associates with another protein through its glycosylated luminal loop and this association could participate in its subcellular trafficking.

A few other clues support that protein–protein interaction events involving luminal portions of lysosomal membrane proteins might promote their targeting to lysosomes. Mutations in the luminal protease-associated domains of RNF13 (A114P) and RNF167 (A104P and V98G) in cancer samples abrogate the endolysosomal localization of these “RING finger proteins” [41]. In other proteins, this conserved protease-associated domain is involved in protein–protein associations [42,43].

#### 2.2.3. Impact of Post-Translational Lipid-Modifications on Lysosomal Membrane Protein Trafficking

As mentioned earlier, the sorting of CLN3 to lysosomes requires an extended dileucine motif (EEEX(8)LI) located in its 2nd cytoplasmic loop, as well as a M(X)9G motif located within the C-terminal region [27,28,32]. Interestingly, an additional level of control is found in a post-translational modification, i.e., the prenylation of a C-terminal CAAX box (C435QLS) [44]. When cysteine 435 is mutated, transport of CLN3 through the endolysosomal system is slowed down.

Similarly, lipid modifications modulate the endolysosomal transport of mucolipin-1, the protein that is deficient in the neurodegenerative lysosomal storage disorder referred to as mucolipidosis IV [45]. Palmitoylation of the C-terminal tail promotes mucolipin internalization from the PM, possibly by reducing the distance between an AP-2 binding signal (E573EHSLL) located further down in the C-terminal region, and the membrane [45]. A second classical dileucine signal (E11TERLL) in the N-terminal region targets mucolipin to lysosomes by an intracellular/direct route, likely AP-1-dependent [45,46]. N-terminal palmitoylation has also been mentioned in [46]. It would be interesting to test whether this modification modulates the presentation of the N-terminal dileucine motif.

The lysosomal sorting of synaptotagmin-7 (i.e., a type I transmembrane protein that regulates lysosomal exocytosis) requires association with the lysosomal membrane protein CD63 (LAMP3) in a palmitoylation-dependent manner [47]. Abrogation of synaptotagmin-7 palmitoylation by mutation of C35, C38 and C41 (located in the transmembrane domain and cytosolic region) prevents association with CD63 and transport to the lysosomes. This transport can also be blocked through mutation of the tyrosine sorting motif in CD63. These findings suggest that CD63 acts as a transport receptor for synaptotagmin-7 and that palmitoylation is a key element of their binding process.

#### 2.2.4. Transmembrane Domain(*S*)-Dependent Sorting of Lysosomal Membrane Proteins

Many groups have reported that transmembrane domains can determine a protein’s subcellular localization and/or control its trafficking within the cell (reviewed by Cosson et al., [48]). Characteristics such as the length of the transmembrane domain, amino acid composition, hydrophobicity, partition into specific lipid-domains, and homo- or heterotypic associations can modulate transport between compartments and favor a given cellular location (ER, Golgi, PM, endosomes, etc.). Regarding lysosomal membrane proteins, one example of transmembrane domain-mediated transport is the homodimeric ATP-binding cassette transporter ABCB6. This protein belongs to a class of long ABC transporters that is characterized by an extended N-terminal domain, referred to as TMD0, which is composed of five transmembrane helices. The TMD0 does not contain classical sorting determinants in its cytosolic portions, but is responsible for the clathrin-dependent internalization and transport of ABCB6 to the endolysosomes [49]. Similarly, the TMD0 of ABCB9 (or the transporter associated with antigen processing-like, TAPL), which only contains four transmembrane helices, comprises the sorting information that drives the protein sorting to the lysosomes [50].

Interestingly, the ABCD4 transporter traffics to the lysosomes when associated with LMBD1 (LMBR1 domain-containing protein 1), a lysosomal protein with nine putative transmembrane domains, that serves as a clathrin adaptor protein for internalization of the insulin receptor [51,52]. The lysosomal sorting of LMBD1 is mediated by a tyrosine-based motif located in a cytosolic loop. When this motif is mutated, both LMBD1 and ABCD4 fail to reach the lysosomes, indicating that ABCD4 makes use of the sorting determinant of another protein to travel to this site. It has been proposed that the interaction between the two proteins requires proper oligomerization of ABCD4, a process that is altered when transmembrane domains 2 and 5 are exchanged with those of ABCD1, a peroxisomal protein [52].

Piggybacking also seems to be the primary mechanism by which the amino acid transport system L is trafficked to the lysosomes. This system is a heterodimer composed of 4F2hc (SLC3A2) and LAT1 (SLC7A5) that localizes at the cell surface as well as in the lysosomes, where it is recruited through association with LAPTM4b, a polytopic lysosomal protein [53]. These proteins associate independently of the cytosolic N- and C-terminal domains of LAPTM4b, possibly already in the ER [53].

#### 2.2.5. Alternatives to the Clathrin-Coated Carriers

The late endosomal and lysosomal membrane proteins LAMP1 and LAMP2 possess a YXXφ signal that mediates their packaging in clathrin-coated vesicles and transport to the lysosomes [11,54,55]. Intriguingly though, several reports have also indicated their presence in non-clathrin-coated carriers. For example, Karlsson and Carlsson documented that LAMP1 and LAMP2 can be packaged at the TGN of HL-60 cells in vesicles devoid of AP-1 and of the cation-independent Man-6-P receptor (CI-MPR), an acid hydrolase receptor that is a cargo of clathrin-coated vesicles [56]. Subsequently, Pols et al. highlighted that non-clathrin-coated vesicles containing LAMP proteins emerge from the TGN and transport their cargo to late endosomes [57]. Using immunoelectron microscopy, they found that these “LAMP carriers” are devoid of CI-MPR, AP-1, and clathrin but contain hVps41, an accessory protein to the vacuolar homotypic fusion and protein sorting (HOPS) complex, as well as the SNARE protein VAMP7. Their findings revealed that these proteins are required for fusion of these carriers with endosomes, thereby discovering a new molecular mechanism (clathrin-independent) by which resident lysosomal membrane proteins can be targeted to their residence site within the cells. Future studies could investigate whether other lysosomal membrane proteins, or hydrolases, use this pathway, and which molecular determinants (sorting motifs, binding sites on adaptor proteins, etc.) control this atypical transport route.

Of note, similar to LAMP1 and 2, LAMP3 (CD63) contains a C-terminal YXXφ motif and is enriched in late endosomes and lysosomes. However, while LAMP1 and -2 mainly localize to the limiting membrane of these organelles, LAMP3 concentrates in the internal vesicles of late endosomes, also referred to as MultiVesicular Bodies (MVBs). The presence of LAMP3 in “LAMP carriers” has not been tested but it has been reported that, in addition to relying on AP-2-dependent endocytosis and on AP-3-dependent sorting from the endosome to move within the cells, LAMP3 can enter the cells by caveolae-mediated endocytosis, i.e., in a clathrin-independent manner [58].

## 3. Subcellular Trafficking of Lysosomal Hydrolases

### 3.1. Mannose 6-Phosphate-Dependent Trafficking

The canonical route of acid hydrolase sorting to the lysosome is referred to as the “mannose 6-phophate (Man-6-P)-dependent pathway” [10,13,15,59,60,61]. During their passage through the Golgi apparatus, most newly synthesized lysosomal acid hydrolases acquire Man-6-P moieties on their N-linked oligosaccharidic chains. These Man-6-P residues serve as recognition signals for two type I transmembrane receptors that transport their ligands to endolysosomes in clathrin-coated carriers: the cation-dependent (CD) and the cation-independent (CI) Man-6-P receptors (MPRs). MPRs contain cytosolic YXXφ, and [D/E]XXXL[L/I] signals that are recognized by several clathrin APs as well as a DXXLL signal that mediates binding to GGAs. However, while the CD-MPR mostly appears to act intracellularly (i.e., in the TGN to endosome sorting), the CI-MPR can mediate hydrolase transport from the TGN as well as from the cell surface, helping to recapture secreted acid hydrolases. Once in the endosomes, the hydrolase-receptor complexes dissociate in the acidic environment, and the receptors recycle to their compartments of origin (PM and/or TGN).

**Figure 1:**
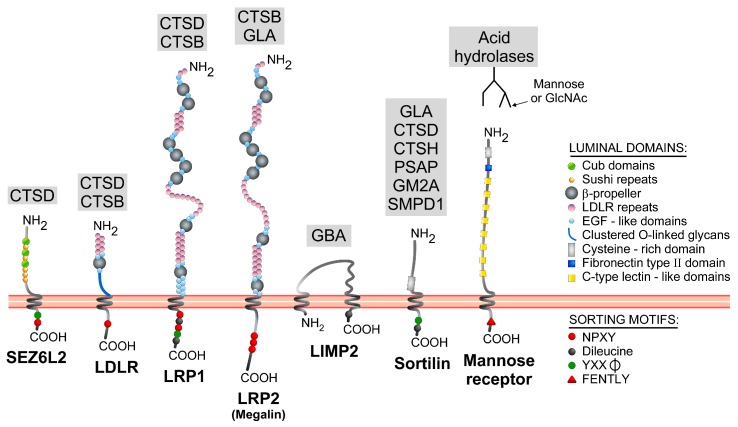
Structure and lysosomal cargo(es) of Man-6-P-alternative receptors. These receptors are mostly type I transmembrane proteins, with the exception of LIMP2, which is type III transmembrane protein. Luminal domains/repeats, and known cytosolic sorting signals are indicated on the scheme. The lysosomal proteins that bind to these receptors are listed in the grey boxes. CTSB: cathepsin B; CTSD: cathepsin D; CTSH: cathepsin H; GLA: α-galactosidase; GBA: β-glucocerebrosidase; PSAP: prosaposin; SMPD1, acid sphingomyelinase; GM2A: Ganglioside GM2 activator protein. The mannose receptor recognizes many acid hydrolases that bear terminal mannose or *N*-acetyl-d-glucosamine on their *N*-linked glycans. All three members of the LDLR family (LDLR, LRP1 and LRP2/Megalin) contain three types of domains in their N-terminal regions: β-propeller, EGF-like repeats and LDLR repeats. The latter are often involved in ligand binding. However, LRP1 binds to cathepsin D (CTSD) through amino acids 349–394 of its β-chain (last 85 kDa of the protein), i.e., a region that contains EGF-like repeats [108]. LIMP2 and β-glucocerebrosidase associate via several hydrophobic helical interfaces located on both proteins [109]. The cysteine-rich domain of sortilin has been involved in binding to several non-lysosomal ligands [110], whereas both the cysteine-rich domain and C-type lectin-like domains 4 and 5 of the mannose receptor serve as ligand binding sites [106]. SEZ6L2 contains Cub domains, which are known to mediate protein–protein interactions.

Man-6-P synthesis is a process mediated by two enzymes that act sequentially: UDP-GlcNAc:lysosomal enzyme *N*-acetylglucosamine-1-phosphotransferase (GlcNAc-1-phosphotransferase) and *N*-acetylglucosamine-1-phosphodiester α-*N*-acetylglucosaminidase (the uncovering enzyme). Mutations in the subunits that compose GlcNAc-1-phosphotransferase give rise to two autosomal recessive disorders: mucolipidosis II (Inclusion-cell disease or I-cell disease) and a less severe pathology, mucolipidosis III (pseudo-Hurler polydystrophy) [62,63]. These disorders are characterized by a defective mannose 6-phosphorylation which results in acid hydrolase hypersecretion and accumulation of non-degraded material in the lysosomal lumen. Consequently, enlarged lysosomes and autolysosomes accumulate within the cells. However, the study of mucolipidosis II and III patients and the characterization of GlcNAc-1-phosphotransferase knock-out and knock-in mice revealed that morphological alterations are only seen in some cell types and tissues, whereas others are able to retain, at least partly, their acid hydrolases and a normal morphology [64,65,66,67,68,69,70,71,72]. Similar observations were made in mice deficient in the two MPRs [73]. All these very intriguing findings suggested very early on (in the 1980s) that alternative(s) to the Man-6-P pathway contribute to the targeting of acid hydrolases to the lysosomes. However, it is mainly in the last 10 years that the most substantial advances have been made in this field with the discovery of several Man-6-P-independent trafficking receptors for acid hydrolases. Table 2 provides a list of such receptors with a summary of their mode of transportation to the endolysosomal system (e.g., direct/indirect trafficking, known sorting motifs, adaptor proteins that recognize these motifs, etc.). Their structures and identified lysosomal cargo(es) are schematized in Figure 1.

### 3.2. Mannose 6-Phosphate-Independent Sorting Receptors

Two of the most well-known alternative MPRs, LIMP2 and Sortilin, have already been discussed in other review articles [13,111,112]. β-glucocerebrosidase is a poorly mannose 6-phosphorylated lysosomal enzyme that is nonetheless transported to the lysosomes, and that exhibits normal or even increased intracellular levels in I-cell disease human and mouse cells [66,68]. It was found that this hydrolase uses LIMP2, a type III lysosomal transmembrane protein, as a transport receptor [113]. Overexpression of LIMP2 causes the enlargement of endosomes and lysosomes and impairs trafficking out of endosomes [114], whereas its deficiency decreases β-glucocerebrosidase activity within the cells and impairs the maturation of acid hydrolases [115]. Consequently, LIMP2 knock-out cells accumulate lipids and exhibit defects in their autophagic and lysosomal degradation pathways, notably reflected by a defective clearance of α-synuclein in the brain [115]. These observations highlight how lysosomal biogenesis relies on LIMP2, which, so far, has only one identified lysosomal hydrolase ligand.

Sortilin is a type I transmembrane protein that can transport several lysosomal proteins from the TGN or PM to the endosomes, including GM2AP (GM2 activator protein), acid sphingomyelinase, prosaposin, as well as cathepsins D and H [85,116,117,118,119,120]. Several of these proteins exhibit normal or near normal levels in some cell types and tissues of I-cell disease patients and mice. However, it is worth noting that the tissues of sortilin knock-out mice that have been examined (brain, liver, lung, testicular tubules, epididymis, efferent ducts, kidney and spleen) exhibit a normal morphology (without signs of lysosomal storage) [118], and that this receptor does not significantly contribute to the endocytosis of cathepsin D in mouse fibroblasts, even when the Man-6-P pathway is disrupted [121]. Hence it appears that lysosomal biogenesis may only rely on sortilin to transport selected acid hydrolases in a subset of cell types, and perhaps only under particular stress conditions (e.g., when the Man-6-P pathway is deficient) [85,116,117,118,120]. For example, it has been reported that, while some amount of cathepsin D and H and acid sphingomyelinase can still be detected in lysosomes of I-cell disease fibroblasts (Man-6-P-deficient), very little intracellular signals remained after overexpression of a truncated sortilin that fails to travel to endosomes, suggesting that sortilin may serve as a Man-6-P-independent endolysosomal transporter when sufficiently expressed in some cells [116,117].

In 2007, Christensen et al. found that α-galactosidase—the lysosomal enzyme that is deficient in Fabry disease—is endocytosed in kidney proximal tubule cells after binding to the cell surface receptor megalin (also named LRP2, low-density lipoprotein receptor-related protein 2), i.e., one of the main receptors involved in the reabsorption of proteins at the kidney proximal tubule [122]. Additionally, this receptor was found to mediate the endocytosis of α-galactosidase in renal podocytes, together with CI-MPR and sortilin [123], and the protease cathepsin B was found secreted and then recaptured via megalin-mediated endocytosis in kidney proximal convoluted tubules [124]. In the latter case, this seems to be a major transport route, followed by cathepsin B, to the lysosomes [124].

In normal fibroblasts, cathepsin B and D can be captured in cells by the cell surface receptors LRP1 (LDL receptor-related protein-1) and LDLR (LDL receptor) [121,125]. It has also been shown that these pathways contribute to the lysosomal sorting of these cathepsins in GlcNAc-1-phosphotransferase knock-in fibroblasts, in which both the LDLR and LRP1 are overexpressed [121]. Indeed, incubation of these Man-6-P-deficient cells with a competitive inhibitor of the LDLR family (the receptor-associated protein, RAP) largely decreases their endocytosis from the extracellular medium [121]. Intriguingly though, internalization and transport to the lysosomes is not the only purpose of these Man-6-P-independent associations. It has been observed that binding of procathepsin D molecules secreted by breast cancer cells to the LRP1 receptor expressed on fibroblasts triggers the fibroblasts outgrowth, and that the mitogenic effect of this hydrolase is independent of its degradation activity [108]. Procathepsin D binds to the extracellular domain of the LRP1 β-chain, an 85 kDa C-terminal fragment that includes several EGF-like domains, the transmembrane segment and the cytosolic portion (Figure 1). Derocq et al. found that the mitogenic effect of procathepsin D derives from the decreased intramembrane proteolytic cleavage of LRP1 when bound to this enzyme [125]. Of note, it has long been known that procathepsin D promotes breast tumor cell proliferation, angiogenesis, and metastasis in an activity-independent manner (reviewed in [126]). In another piece of relevant research, this enzyme was found to mostly be endocytosed by a Man-6-P-independent mechanism in breast cancer cell lines [127]. However, whether LRP1 is the only transport receptor involved in the internalization and paracrine/autocrine effect of procathepsin D on tumor cells remains to be seen.

Another example of a Man-6-P-independent receptor that plays a role in cellular regulation, in addition to providing a transportation mean to endolysosomes is the protein SEZ6L2. Our lab recently identified that this type I transmembrane protein predominantly expressed in brain can participate in the subcellular trafficking of procathepsin D to endosomes in neuronal cells [105]. Interestingly, the cleavage of SEZ6L2 by this protease in N1E-115 neuronal cells, possibly upon arrival in endosomes, generates an N-terminal soluble fragment that induces neurite outgrowth, while its membrane counterpart prevents this. As SEZ6L2 is also cleaved by the aspartic proteases BACE1 and -2 (β-secretases) in neurons and pancreatic β-cells, respectively [128,129], it could prove valuable to investigate the putative involvement of SEZ6L2 in the subcellular trafficking of these enzymes. In addition, due to their sequence homology, a next step could be to determine whether the two other members of the SEZ6 protein family (SEZ6 and SEZ6L) can also transport cathepsin D (or other lysosomal proteins), especially since their deficiency has been associated with an alteration in neuronal branching as well [130,131].

It is also worth mentioning that new lessons have been learned about an “old Man-6-P alternative receptor” in the past ten years. It has been long known that a circulating acid hydrolase that bears an oligosaccharidic chain terminated by *N*-acetylglucosamine or mannose residues can be rapidly internalized in liver endothelial cells and, to a lesser extent, in macrophages [132,133,134]. The receptor responsible for this endocytosis process is a type I transmembrane protein of 175 kDa with a cytosolic C-terminal end, termed the “mannose receptor” [135,136]. Surprisingly, despite the many reports of its involvement in the endocytosis of radiolabeled acid hydrolases in vitro and in vivo, the relative contribution of this receptor to lysosome biogenesis in basal conditions has not been easy to assess. While the levels of several acid hydrolases increase in the serum of mice with a knocked-out mannose receptor gene, it has been reported that total endogenous acid hydrolase levels in the heart, spleen, liver, lung, and kidney remain relatively unchanged [137]. It is only when the focus was set on cells that express high levels of this receptor that it became obvious that the mannose receptor can significantly contribute to the acquisition of the lysosomal hydrolytic arsenal. Elvevold et al. estimated that the lysosomal degradation activity of liver sinusoidal cells decreases by almost 50 percent after knock-down of the mannose receptor [138]. Moreover, activity assays of several lysosomal hydrolases (cathepsin D, α-mannosidase, β-hexosaminidase, and arylsulfatase) highlighted a 68%–97% decrease of their total intracellular levels in these knock-out cells, indicating that the bulk of the lysosomal hydrolase population is obtained through mannose receptor-dependent capture of extracellular enzymes in non-parenchymal cells of the liver.

Another interesting finding in regards to the mannose receptor is that it can also be used by poorly mannose 6-phosphorylated acid hydrolases to reach the lysosomes. The hyaluronidase HYAL1 is a highly secreted enzyme that has never been found in the Man-6-P proteome [6,139], even though its acidic optimum pH suggested that it would not be active outside of the endolysosomal system. Our recent study of the subcellular trafficking of this glycoprotein resolved this apparent discrepancy by showing that, despite its very low level of mannose 6-phosphorylation, HYAL1 accumulates in the lysosomes of a mouse macrophage cell line, as well as in liver lysosomes (likely in non-parenchymal cells) [140,141]. The mannose receptor mediates the recapture of HYAL1 from the medium where it is largely exported by constitutive secretion. Importantly, we found that the intralysosomal level of HYAL1 is largely controlled by this recapture mechanism, indicating that this can be a major trafficking pathway to the lysosome for poorly phosphorylated hydrolases. Actually, one of the enzyme-replacement strategies used to treat Gaucher disease is based on the modification of the oligosaccharidic chains of β-glucocerebrosidase, which does not acquire Man-6-P signals either, to expose more mannose residues and facilitate its mannose receptor-mediated internalization in macrophages, which are strongly affected by this enzyme deficiency [142,143].

Of note, we also found that HYAL1 localizes to lysosomes in osteoclasts, the giant bone resorbing cells that differentiate from the monocyte/macrophage lineage. However, in these cells, secretion/recapture by the mannose receptor was found to be inefficient compared to macrophages, and the Man-6-P pathway did not significantly contribute to HYAL1 sorting to lysosomes either [144]. Similarly, the transport of the newly found lysosomal enzyme arylsulfatase G appears to be independent of the MPRs, as well as of sortilin and LIMP2 in mouse liver [145]. There are also reports of the Man-6-P-independent trafficking of acid α-glucosidase by an unknown, Man-6-P-independent mechanism [146]. These observations indicate that much is left to learn on the subcellular trafficking mechanisms that control the sorting of lysosomal enzymes independently of the Man-6-P signal. According to Markmann et al., who compared the relative levels of acid hydrolases in control fibroblasts and fibroblasts deficient for GlcNAc-1-phosphotransferase (i.e., unable to synthesize Man-6-P) by quantitative mass spectrometry, close to 20% of the enzymes (11 out of 51 found in the samples) were efficiently retained in the cells after disruption of the Man-6-P pathway [121]. Thus these proteins are likely the cargoes of Man-6-P-independent receptors, as supported by the presence among these proteins of several of the enzymes cited above (cathepsin D, cathepsin B, β-glucocerebrosidase, GM2AP, prosaposin, etc.) as well as a few others for which alternative receptors have yet to be identified.

An interrogation that also remains unanswered is the relative contribution of each Man-6-P alternative receptors to the sorting of their respective hydrolase ligand(s) in I-cell disease. For example, it has been clearly shown that, despite the absence of Man-6-phosphorylation, procathepsin D is largely retained in several cell types and tissues of I-cell disease patients and mouse models, including lymphoblasts, osteoclasts, pancreas, brain, liver and salivary glands [67,68,69,71]. However, it is unclear to what extent the different Man-6-P-independent cathepsin D receptors (LDLR, LRP1, LRP2, SEZ6L2, sortilin and mannose receptor) participate in its sorting in all of these tissues. Knowing that some of these receptors have distinct expression profile (e.g., SEZ6L2 is located in the brain, the mannose receptor is mainly found in endothelial and macrophage-type cells, LRP2 in the kidneys), some tissue-specificity of Man-6-P alternative pathways is to be expected.

## 4. Acquisition of Resident Lysosomal Proteins from the Cytosol

The lysosomal proteome is composed of transmembrane proteins and acid hydrolases, but also includes many “lysosome-associated proteins”, i.e., proteins that bind to the external leaflet of the lysosomal membrane. These proteins can be recruited from the cytosol by very diverse mechanisms.

For example, the Rab GTPase Rab9 is recruited on late endosomes through interaction with the cytosolic protein TIP47, a cytosolic effector protein that binds to the MPRs’ tails [147,148].

The multiprotein complex mTORC1, which regulates cellular growth and metabolism, localizes in the cytosol under low amino acids conditions, and is recruited on the lysosomal membrane when amino acids are abundant [149,150]. This recruitment is controlled by a vATPase–Ragulator–Rag GTPases complex. When the GTPases are in an inactive conformation (at low amino acid levels), mTORC1 remains in the cytosol. By contrast, when amino acid levels are high, they trigger the activation of the lysosomal RabGTPases, which recruit mTORC1 on the lysosomal membrane. Amino acids also control the assembly of the peripheral vATPase domain (V1) to its integral membrane domain (V0), and thereby modulate the proton transport activity of the pump, as well as its association with the ragulator complex and activation of mTORC1 [151,152].

It has also been documented that three proteins associated with cases of hereditary spastic paraplegia (SPG11/spatacsin, SPG15/spastizin, and SPG48/AP-5) are recruited as a complex on the surface of lysosomes and autolysosomes (autophagolysosomes) [153]. The complex may use spastizin as an anchor on the membrane, as it has been documented that this protein interacts with PI3P via its FYVE domain [154]. Interestingly, fibroblasts isolated from SPG11, SPG15 or SPG48 patients, all contain enlarged late endosomes/lysosomes [18,155], and, in the context of macroautophagy, it has been shown that SPG11 and 15 are required for the reformation of lysosomes from autolysosomes by a process that involves tubulation from the autolysosomes [154].

These findings clearly support that lysosome-associated proteins are crucial to lysosomal function.

## 5. New Directions

### 5.1. Is There a Biosynthetic Trafficking Route to the Endolysosomes That Does Not Require Passage through the Golgi Apparatus?

It is commonly accepted that lysosomal transmembrane proteins and acid hydrolases are synthesized in the ER and that they follow the classical secretory pathway to the lysosomes (which includes passage through the Golgi apparatus). However, the identification of what is referred to as “Golgi-bypassing routes” and “unconventional secretory pathways” for several non-lysosomal proteins (reviewed in [156,157,158]) suggests that there might be additional ways to reach the lysosomes. For example, it has been found that several cell surface transmembrane proteins may use, in addition to the classical secretory pathway, a route that does not pass through the Golgi apparatus to reach the PM. Additionally, there are several examples of soluble nuclear/cytosolic proteins (i.e., that lack signal peptides and hence are unable to enter the classical secretory pathway) that are nonetheless excreted in the extracellular space. One proposal is that the sorting mechanism to the cell surface includes transport from the ER or ER-Golgi Intermediate Compartment (for membrane proteins), or from the cytosol (for soluble proteins), to autophagic or endolysosomal structures that would then fuse with the PM. Direct transport from the ER to the cell surface has also been suggested for membrane proteins. These unconventional routes have notably been inferred from the identification of key molecular players, including GRASPs (Golgi reassembly stacking proteins), Syntaxins, which control endosomal trafficking, and some members of the autophagy-related protein (ATG) family (as reviewed in [156,157,158,159]). What is noteworthy here is that some cellular stresses, such as ER stress and autophagy induction, may increase the proportion of proteins traveling via these atypical pathways.

Even though the clearance of proteins through lysosomal degradation or cellular export might be the main function of some of these Golgi-bypassing/unconventional secretion routes, we believe that it is worth considering that the lysosomes might acquire some proportion of their resident proteins through those pathways, in normal or stress conditions. We recently found that low amounts of ATG9A, a transmembrane protein that resides in endosomes and the TGN under basal conditions [160], travels to the cell surface via a Golgi-bypassing route [161]. Moreover, when the transport of ATG9A through the Golgi stacks is prevented by mutation of a LYM motif located in its C-terminal region, the Golgi-bypassing route is sufficient to supply normal intracellular levels of ATG9A to the endosomes [161]. These observations support that the Golgi-bypassing pathway can be a very efficient way to target transmembrane proteins to endolysosomes. Regarding soluble proteins, as the classical examples that use the unconventional secretory pathway are cytosolic/nuclear proteins devoid of signal peptides, it is tempting to posit that lysosomal hydrolases, which contain signal peptides and are synthesized in the ER, would be poor candidates for sorting by a Golgi-bypassing route. However, the extracellular proteoglycan serglycin, which contains a signal peptide, can still exit from the cells when transport from the ER to the Golgi is inhibited, or when the protein is fused to a KDEL (lysine, aspartic acid, glutamic acid, leucine) ER retention sequence that prevents its transport through the Golgi apparatus [162]. Hence lysosomal hydrolases may well be able to bypass the Golgi on their way to the endolysosomes or the extracellular space, from where they could eventually be recaptured. Further work will be needed to test these hypotheses, as well as to assess whether lysosomal transmembrane or soluble proteins originating from the ER have sufficient time to fold properly prior to their entry in the Golgi-bypassing route, and to analyze how their immature glycosylation pattern affects their stability and function in endolysosomes.

### 5.2. Acquisition of the Lysosomal Proteome of Another Cell

Even though the benefits of hematopoietic stem cell (HSC) transplantation for the treatment of non-hematopoietic diseases are sometimes called into question, Cherqui and Courtois found that this treatment can efficiently correct thyroid, kidney and eye dysfunctions in a mouse model of cystinosis, an inherited lysosomal storage disease caused by a cystinosin deficiency [163,164,165]. Quite surprisingly, they observed that transplanted HSCs differentiate into macrophages that extend “tunneling nanotube-like structures” towards cystinosin-deficient cells types, including thyrocytes, kidney tubular cells, fibroblasts and eye corneal cells [164,165,166]. These nanotube-like structures are the basis of a cross-correction process, i.e., they allow for the bidirectional transport of lysosomes between the connected cells, hence providing the cystinosin-deficient cells with fully functional lysosomes. In vitro testing revealed that nanotube-formation and lysosome-exchange events were more numerous when the macrophages were co-cultured with cystinosin-deficient fibroblasts than with wild-type fibroblasts, suggesting that stress conditions might serve as a trigger. However, knowing that nanotube-like extensions have been detected between other cell types in basal conditions [167,168], it would be very interesting to test whether this cell-to-cell transfer organelle system can significantly contribute to the lysosomal digestive capacity of some cells.

Lastly, cells shed microvesicles in the extracellular medium either from their PM (ectosomes), or by exocytosis of the microvesicles contained within multi-vesicular endosomes (exosomes, which bud and detach from the endosomal limiting membrane) [169]. After release in the extracellular space, these microvesicles can be endocytosed by clathrin-mediated and clathrin-independent internalization, micropinocytosis and phagocytosis, notably by neighboring cells. In 2012, it was documented that mesenchymal stem cells shed microvesicles that contain wild-type cystinosin and cystinosin-encoding RNA, and that the incorporation of these vesicles in cystinosin deficient fibroblasts or proximal tubular cells in vitro decreases their abnormal accumulation of cystine [170]. As other lysosomal proteins, including LAMP3, have also been detected in shedded microvesicles (Exocarta database, [171]), this process is worth considering in a lysosomal biogenesis perspective.

## 6. Concluding Remarks

**Figure 2:**
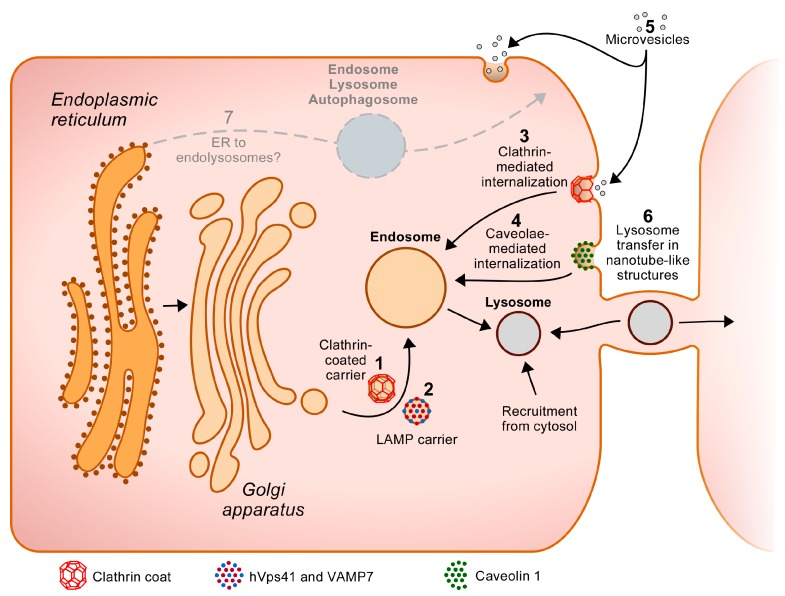
Sorting pathways to the endolysosomes. Newly synthesized lysosomal proteins are transported to the Golgi apparatus from where they reach the endosomes and, subsequently the lysosomes. One of the main transport pathways followed by these proteins is a direct route from the TGN to the endosomes (**1**). The lysosomal transmembrane proteins and acid hydrolase receptors (MPRs, sortilin, SEZ6L2) that are bound to their ligands are packaged at the TGN in clathrin-coated carriers that travel toward the endosomes. This packaging is driven by the binding of typical cytosolic tyrosine- or dileucine-based sorting signals, or of atypical motifs, to clathrin adaptor proteins (directly or through some other proteins). Alternatively, some transmembrane proteins (including LAMP1 and LAMP2) can be transported directly from the TGN to the endosomes in non-clathrin-coated vesicles (“LAMP carriers”) that are positive for hVps41 and VAMP7 (**2**). Lysosomal proteins that are not packaged in transport vesicles at the TGN, and escape to the cell surface as a consequence, can be recaptured by clathrin-mediated internalization, which is also based on the recognition of cytosolic sorting motifs by PM clathrin adaptor proteins (**3**). In addition, LAMP3 has been found to enter the endolysosomal system by caveolin-mediated internalization (**4**). Of note, some lysosomal proteins may piggyback on others to enter some of these pathways (as indicated in Table 1). The cells can also acquire some lysosomal proteins from other cells, e.g., through the capture of microvesicles by clathrin-dependent and -independent mechanisms (**5**) or subsequently to the transfer of exogenous lysosomes through nanotube-like structures tunneling between some cells (**6**). Lastly, some proportion of lysosomal proteins could, hypothetically, bypass the Golgi apparatus to reach endolysosomes or autophagosomes (which could then fuse with lysosomes) directly from the ER, or after sorting from the ER to the PM and subsequent internalization from this site (**7**).

The wide array of subcellular trafficking mechanisms and routes that mediate the sorting of lysosomal proteins to their residence site in the cells speaks to very complex molecular interplays that have to take place to achieve normal lysosomal functions. The pathways discussed in this review have been summarized in Figure 2. It should be noted that not all pathways may be active in all cell types. Classical MPRs exhibit slightly different ligand binding properties [172], as well as differences in their tissue expression patterns [173,174,175]. Furthermore, some alternative MPRs have very restricted cell expression profiles and, while clathrin adaptor protein subunits, coat proteins of “LAMP carriers” (Vps41 and VAMP7), GRASPs, ATG proteins, and many other players of conventional and non-conventional transport pathways are expressed in most organs, their expression levels can sometimes differ between cell types (see the Human Protein Atlas, available from www.proteinatlas.org [176]). Additional investigations will therefore be needed to assess the relative contribution of conventional versus non-conventional transport mechanisms to the endolysosomes in different cell types. How some atypical sorting motifs can mediate endolysosomal sorting, and what the extent and role is of Man-6-P-independent transport mechanisms in lysosomal function and dysfunctions are also questions that remain open.

## Figures and Tables

**Table 1:** Lysosomal transmembrane proteins with atypical sorting signals or transport routes to the lysosomes.

Gene Symbol	Protein Name	Conventional Sorting Determinant(s)	Atypical Sorting Determinant(s)	Trafficking Mechanism(s)	References
*CTNS*	Cystinosin	GYDQL in C-ter tail	YFPQA in 3rd cytoplasmic loop	AP-3 -dependent intracellular/direct trafficking.	[19,20]
*VAMP7*	Vesicle-associated membrane protein 7		N-ter longin domain (critical residues: Leu^43^/Tyr^45^)	*cis*-SNARE complex transported by AP-3-dependent direct trafficking, and by Hrb-dependent endocytosis.	[21,22,23]
*LAPTM5*	Lysosomal-associated transmembrane protein 5		PY motifs (L/PPxY) + ubiquitin-interacting motif (LKVALPSYEE)	PY motifs recruits GGA3, which binds to the ubiquitin-interacting motif of LAPTM5 and mediates transport to endolysosomes.	[24]
*LAPTM4A*	Lysosomal-associated transmembrane protein 4A	YXXφ motif in C-ter region	PY motifs in C-ter tail	Nedd4-dependent sorting to endolysosomes.	[25,74]
*LAPTM4B*	Lysosomal-associated transmembrane protein 4B		PY motifs in C-ter tail	Nedd4-dependent sorting to endolysosomes.	[25]
*CLN3*	Battenin		Atypical dileucine motif (EEEX(8)LI) in a cytoplasmic loop; MX9G in C-ter tail; C-ter CAAX farnesylation motif (C^435^QLS)	Mostly AP-1 and AP-3-mediated intracellular sorting. Inhibition of farnesylation induces relocalization to the PM and slows transport to endolysosomes.	[27,28,32,44]
*TMEM106B*	Transmembrane protein 106B		Extended dileucine motif (ENQLVALI) in the N-ter region; 4th and 5th *N*-glycosylation sites	Mutation of the 4th and 5th *N*-glycosylation sites results in ER retention and relocalization at the PM, respectively. Mutation of LI in the atypical dileucine signal results in a diffuse cytoplasmic localization.	[26,40]
*GRP143/OA1*	G-protein coupled receptor 143		Unconventional dileucine motif (SLLKGRQGIY) in the 3rd cytosolic loop; WE (tryptophan/Glutamic acid) motif in C-ter tail		[33]
*STARD3*	StAR-related lipid transfer protein 3/MLN64 (metastatic lymph node 64)		14-3-3 binding motif (K392SASNP) in the START domain (C-ter); unidentified internalization motif in the N-ter cytosolic region or transmembrane domains	Indirect trafficking via PM. Mutation of the 14-3-3 binding site delays transport to endosomes via the cell surface.	[34,39]
*RNF13*	E3 ubiquitin-protein ligase RNF13 (Ring finger protein 13)		Luminal protease-associated domain	A114P substitution in the luminal protease-associated domain prevents sorting to endolysosomes.	[41]
*RNF167*	E3 ubiquitin-protein ligase RNF167 (Ring finger protein 167)		Luminal protease-associated domain	A104P and V98G substitutions in the luminal protease-associated domain prevent sorting to endolysosomes.	[41]
*MCOLN1*	Mucolipin-1	ETERLL in N-ter domain; EEHSLL in C-ter domain	Cysteines 565–567	ETERLL-mediated direct transport, likely mediated by AP-1. EEHSLL-mediated internalization, AP-2–mediated. Palmitoylation of cysteines 565–567 promotes internalization, possibly by bringing the C-ter dileucine signal closer to the membrane.	[45,46]
*CD63*	CD63 antigen/LAMP3	GYEVM in C-ter region		Direct and indirect transport. C-ter domain binds to AP-2, AP-3, and AP-4. Internalization from the cell surface via caveolae.	[58,75,76,77,78]
*SYT7*	Synaptotagmin-7		Cysteines 35, 38 and 41 close to and in the transmembrane domain	Palmitoylation-dependent piggybacking on CD63.	[47]
*ABCB6*	ATP-binding cassette subfamily B member 9		Extended N-ter domain (TMD0) which contains 5 transmembrane helices	Clathrin-dependent internalization.	[49]
*ABCB9*	ATP-binding cassette subfamily B member 9		TMD0, composed of four transmembrane helices		[50]
*ABCD4*	ATP-binding cassette subfamily D member 4		Possibly transmembrane domains 2 and 5	Clathrin-dependent internalization. Piggybacking on LMBD1, which uses a Yxxφ sorting signal.	[51,52]
*LAMP1*	Lysosome-associated membrane glycoprotein 1	C-ter GYQTI		AP-1- and AP-3-dependent direct sorting. AP-2-dependent internalization. Sorted in vesicles positive for hVps41 and VAMP7, negative for CI-MPR, AP-1 and clathrin.	[54,55,56,57,75,78,79,80,81,82,83]
*LAMP2*	Lysosome-associated membrane glycoprotein 2	C-ter YEQF		AP-1-and AP-3-dependent direct sorting. AP-2-dependent internalization. Binds to AP-4. Sorted in vesicles positive for hVps41 and VAMP7, negative for CI-MPR, AP-1 and clathrin.	[56,57,75,78,82,84]
*SLC3A2/SLC7A5*	4F2hc/LAT1			Piggybacking on LAPTM4b.	[53]

**Table 2:** Alternatives to the mannose 6-phosphate receptors.

Gene Name	Protein Name	Endosomal Sorting Motif(s)	Endosomal Sorting Mechanism(s)	References
*SORT1*	Sortilin	YXXφ (YSVL) and dileucine (DDSDEDLI) signals in C-ter region	Direct route: GGA-mediated (dileucine); AP-1-mediated (YXXφ). Indirect route: Clathrin-dependent internalization (YXXφ).	[85,86,87,88,89]
*SCARB2*	LIMP2	Dileucine signal (DERAPLI) in C-ter region	Direct route: AP-1 and AP-3-mediated via the dileucine motif. Indirect route: minor in some cell types.	[90,91,92,93,94,95]
*LDLR*	LDL (low-density lipoprotein) receptor	NPXY signal (NPVY) in the C-ter tail	Indirect route: NPXY- and ARH-dependent internalization. Binding to AP-2 reported.	[96,97,98,99]
*LRP1*	LDL receptor-related protein 1	YXXφ (YATL), NPXY (NPTY and NPVY), dileucine (DDVGGLL and DEKRELL) signals in C-ter region	Indirect route: mediated by YXXφ and dileucine motifs closest to the C-ter end. Minor involvement of the other signals. Binding to AP-2 and clathrin reported.	[100,101]
*LRP2*	Megalin/low-density lipoprotein receptor-related protein 2	NPXY signals in the C-ter region	Indirect route: NPXY-dependent internalization. Proximal NPXY binds to ARH; distal NPXY binds to Dab2.	[102,103,104]
*SEZ6L2*	Seizure 6-like protein 2/Brain Specific Receptor-like Protein A (BSRP-A)	YXXφ (YSPI) and NPXY (NPLY) signals in the C-ter region	Direct route: likely YXXφ -mediated; SEZ6L2 detected in AP-1 positive clathrin-coated vesicles. Indirect route: NPXY- and Dab-2 mediated internalization.	[105]
*MRC1*	Mannose receptor	FENTLY in the C-ter domain	Indirect route: Transmembrane domain and FENTLY-dependent internalization.	[106,107]

## References

[1] De Duve C., Pressman B.C., Gianetto R., Wattiaux R., Appelmans F. (1955). Tissue fractionation studies. 6. Intracellular distribution patterns of enzymes in rat-liver tissue. Biochem. J..

[2] Sabatini D.D., Adesnik M. (2013). Christian de Duve: Explorer of the cell who discovered new organelles by using a centrifuge. Proc. Natl. Acad. Sci. USA.

[3] Schröder B., Wrocklage C., Pan C., Jäger R., Kösters B., Schäfer H., Elsässer H.-P., Mann M., Hasilik A. (2007). Integral and associated lysosomal membrane proteins. Traffic (Cph. Den.).

[4] Callahan J.W., Bagshaw R.D., Mahuran D.J. (2009). The integral membrane of lysosomes: Its proteins and their roles in disease. J. Proteom..

[5] Della Valle M.C., Sleat D.E., Zheng H., Moore D.F., Jadot M., Lobel P. (2011). Classification of subcellular location by comparative proteomic analysis of native and density-shifted lysosomes. Mol. Cell. Proteom. MCP.

[6] Sleat D.E., Sun P., Wiseman J.A., Huang L., El-Banna M., Zheng H., Moore D.F., Lobel P. (2013). Extending the mannose 6-phosphate glycoproteome by high resolution/accuracy mass spectrometry analysis of control and acid phosphatase 5-deficient mice. Mol. Cell. Proteom. MCP.

[7] Chapel A., Kieffer-Jaquinod S., Sagné C., Verdon Q., Ivaldi C., Mellal M., Thirion J., Jadot M., Bruley C., Garin J. (2013). An extended proteome map of the lysosomal membrane reveals novel potential transporters. Mol. Cell. Proteom. MCP.

[8] Ballabio A., Gieselmann V. (2009). Lysosomal disorders: From storage to cellular damage. Biochim. Biophys. Acta.

[9] Platt F.M., Boland B., van der Spoel A.C. (2012). The cell biology of disease: Lysosomal storage disorders: The cellular impact of lysosomal dysfunction. J. Cell Biol..

[10] Ghosh P., Dahms N.M., Kornfeld S. (2003). Mannose 6-phosphate receptors: New twists in the tale. Nat. Rev. Mol. Cell Biol..

[11] Bonifacino J.S., Traub L.M. (2003). Signals for sorting of transmembrane proteins to endosomes and lysosomes. Annu. Rev. Biochem..

[12] Robinson M.S. (2004). Adaptable adaptors for coated vesicles. Trends Cell Biol..

[13] Braulke T., Bonifacino J.S. (2009). Sorting of lysosomal proteins. Biochim. Biophys. Acta.

[14] Robinson M.S. (2015). Forty years of Clathrin-coated vesicles. Traffic (Cph. Den.).

[15] Kollmann K., Pohl S., Marschner K., Encarnação M., Sakwa I., Tiede S., Poorthuis B.J., Lübke T., Müller-Loennies S., Storch S. (2010). Mannose phosphorylation in health and disease. Eur. J. Cell Biol..

[16] Hirst J., Irving C., Borner G.H.H. (2013). Adaptor protein complexes AP-4 and AP-5: New players in endosomal trafficking and progressive spastic paraplegia. Traffic (Cph. Den.).

[17] Saftig P., Klumperman J. (2009). Lysosome biogenesis and lysosomal membrane proteins: Trafficking meets function. Nat. Rev. Mol. Cell Biol..

[18] Hirst J., Edgar J.R., Esteves T., Darios F., Madeo M., Chang J., Roda R.H., Dürr A., Anheim M., Gellera C. (2015). Loss of AP-5 results in accumulation of aberrant endolysosomes: Defining a new type of lysosomal storage disease. Hum. Mol. Genet..

[19] Cherqui S., Kalatzis V., Trugnan G., Antignac C. (2001). The targeting of cystinosin to the lysosomal membrane requires a tyrosine-based signal and a novel sorting motif. J. Biol. Chem..

[20] Andrzejewska Z., Névo N., Thomas L., Bailleux A., Chauvet V., Benmerah A., Antignac C. (2015). Lysosomal targeting of cystinosin requires AP-3. Traffic (Cph. Den.).

[21] Pryor P.R., Jackson L., Gray S.R., Edeling M.A., Thompson A., Sanderson C.M., Evans P.R., Owen D.J., Luzio J.P. (2008). Molecular basis for the sorting of the SNARE VAMP7 into endocytic clathrin-coated vesicles by the ArfGAP Hrb. Cell.

[22] Kent H.M., Evans P.R., Schäfer I.B., Gray S.R., Sanderson C.M., Luzio J.P., Peden A.A., Owen D.J. (2012). Structural basis of the intracellular sorting of the SNARE VAMP7 by the AP3 adaptor complex. Dev. Cell.

[23] Martinez-Arca S., Rudge R., Vacca M., Raposo G., Camonis J., Proux-Gillardeaux V., Daviet L., Formstecher E., Hamburger A., Filippini F. (2003). A dual mechanism controlling the localization and function of exocytic v-SNAREs. Proc. Natl. Acad. Sci. USA.

[24] Pak Y., Glowacka W.K., Bruce M.C., Pham N., Rotin D. (2006). Transport of LAPTM5 to lysosomes requires association with the ubiquitin ligase Nedd4, but not LAPTM5 ubiquitination. J. Cell Biol..

[25] Milkereit R., Rotin D. (2011). A role for the ubiquitin ligase Nedd4 in membrane sorting of LAPTM4 proteins. PLoS ONE.

[26] Busch J.I., Unger T.L., Jain N., Tyler Skrinak R., Charan R.A., Chen-Plotkin A.S. (2016). Increased expression of the frontotemporal dementia risk factor TMEM106B causes C9orf72-dependent alterations in lysosomes. Hum. Mol. Genet..

[27] Storch S., Pohl S., Braulke T. (2004). A dileucine motif and a cluster of acidic amino acids in the second cytoplasmic domain of the batten disease-related CLN3 protein are required for efficient lysosomal targeting. J. Biol. Chem..

[28] Kyttälä A., Ihrke G., Vesa J., Schell M.J., Luzio J.P. (2004). Two motifs target Batten disease protein CLN3 to lysosomes in transfected nonneuronal and neuronal cells. Mol. Biol. Cell.

[29] Nicholson A.M., Rademakers R. (2016). What we know about TMEM106B in neurodegeneration. Acta Neuropathol..

[30] Cárcel-Trullols J., Kovács A.D., Pearce D.A. (2015). Cell biology of the NCL proteins: What they do and don’t do. Biochim. Biophys. Acta.

[31] Sleat D.E., Gedvilaite E., Zhang Y., Lobel P., Xing J. (2016). Analysis of large-scale whole exome sequencing data to determine the prevalence of genetically-distinct forms of neuronal ceroid lipofuscinosis. Gene.

[32] Kyttälä A., Yliannala K., Schu P., Jalanko A., Luzio J.P. (2005). AP-1 and AP-3 facilitate lysosomal targeting of Batten disease protein CLN3 via its dileucine motif. J. Biol. Chem..

[33] Piccirillo R., Palmisano I., Innamorati G., Bagnato P., Altimare D., Schiaffino M.V. (2006). An unconventional dileucine-based motif and a novel cytosolic motif are required for the lysosomal and melanosomal targeting of OA1. J. Cell Sci..

[34] Liapis A., Chen F.W., Davies J.P., Wang R., Ioannou Y.A. (2012). MLN64 transport to the late endosome is regulated by binding to 14-3-3 via a non-canonical binding site. PLoS ONE.

[35] Muslin A.J., Xing H. (2000). 14-3-3 proteins: Regulation of subcellular localization by molecular interference. Cell Signal..

[36] Martina J.A., Chen Y., Gucek M., Puertollano R. (2012). MTORC1 functions as a transcriptional regulator of autophagy by preventing nuclear transport of TFEB. Autophagy.

[37] Smith A.J., Daut J., Schwappach B. (2011). Membrane proteins as 14-3-3 clients in functional regulation and intracellular transport. Physiology (Bethesda).

[38] Efendiev R., Chen Z., Krmar R.T., Uhles S., Katz A.I., Pedemonte C.H., Bertorello A.M. (2005). The 14-3-3 protein translates the NA^+^,K^+^-ATPase α1-subunit phosphorylation signal into binding and activation of phosphoinositide 3-kinase during endocytosis. J. Biol. Chem..

[39] Zhang M., Liu P., Dwyer N.K., Christenson L.K., Fujimoto T., Martinez F., Comly M., Hanover J.A., Blanchette-Mackie E.J., Strauss J.F. (2002). MLN64 mediates mobilization of lysosomal cholesterol to steroidogenic mitochondria. J. Biol. Chem..

[40] Lang C.M., Fellerer K., Schwenk B.M., Kuhn P.-H., Kremmer E., Edbauer D., Capell A., Haass C. (2012). Membrane orientation and subcellular localization of transmembrane protein 106B (TMEM106B), a major risk factor for frontotemporal lobar degeneration. J. Biol. Chem..

[41] Van Dijk J.R., Yamazaki Y., Palmer R.H. (2014). Tumour-associated mutations of PA-TM-RING ubiquitin ligases RNF167/RNF13 identify the PA domain as a determinant for endosomal localization. Biochem. J..

[42] Luo X., Hofmann K. (2001). The protease-associated domain: A homology domain associated with multiple classes of proteases. Trends Biochem. Sci..

[43] Luo F., Fong Y.H., Zeng Y., Shen J., Jiang L., Wong K.-B. (2014). How vacuolar sorting receptor proteins interact with their cargo proteins: Crystal structures of apo and cargo-bound forms of the protease-associated domain from an Arabidopsis vacuolar sorting receptor. Plant Cell.

[44] Storch S., Pohl S., Quitsch A., Falley K., Braulke T. (2007). C-terminal prenylation of the CLN3 membrane glycoprotein is required for efficient endosomal sorting to lysosomes. Traffic (Cph. Den.).

[45] Vergarajauregui S., Puertollano R. (2006). Two di-leucine motifs regulate trafficking of mucolipin-1 to lysosomes. Traffic (Cph. Den.).

[46] Miedel M.T., Weixel K.M., Bruns J.R., Traub L.M., Weisz O.A. (2006). Posttranslational cleavage and adaptor protein complex-dependent trafficking of mucolipin-1. J. Biol. Chem..

[47] Flannery A.R., Czibener C., Andrews N.W. (2010). Palmitoylation-dependent association with CD63 targets the Ca^2+^ sensor synaptotagmin VII to lysosomes. J. Cell Biol..

[48] Cosson P., Perrin J., Bonifacino J.S. (2013). Anchors aweigh: Protein localization and transport mediated by transmembrane domains. Trends Cell Biol..

[49] Kiss K., Kucsma N., Brozik A., Tusnady G.E., Bergam P., van Niel G., Szakacs G. (2015). Role of the N-terminal transmembrane domain in the endo-lysosomal targeting and function of the human ABCB6 protein. Biochem. J..

[50] Demirel O., Bangert I., Tampé R., Abele R. (2010). Tuning the cellular trafficking of the lysosomal peptide transporter TAPL by its N-terminal domain. Traffic (Cph. Den.).

[51] Tseng L.T.-L., Lin C.-L., Tzen K.-Y., Chang S.C., Chang M.-F. (2013). LMBD1 protein serves as a specific adaptor for insulin receptor internalization. J. Biol. Chem..

[52] Kawaguchi K., Okamoto T., Morita M., Imanaka T. (2016). Translocation of the ABC transporter ABCD4 from the endoplasmic reticulum to lysosomes requires the escort protein LMBD1. Sci. Rep..

[53] Milkereit R., Persaud A., Vanoaica L., Guetg A., Verrey F., Rotin D. (2015). LAPTM4b recruits the LAT1–4F2hc Leu transporter to lysosomes and promotes mTORC1 activation. Nat. Commun..

[54] Williams M.A., Fukuda M. (1990). Accumulation of membrane glycoproteins in lysosomes requires a tyrosine residue at a particular position in the cytoplasmic tail. J. Cell Biol..

[55] Höning S., Griffith J., Geuze H.J., Hunziker W. (1996). The tyrosine-based lysosomal targeting signal in lamp-1 mediates sorting into Golgi-derived clathrin-coated vesicles. EMBO J..

[56] Karlsson K., Carlsson S.R. (1998). Sorting of lysosomal membrane glycoproteins lamp-1 and lamp-2 into vesicles distinct from mannose 6-phosphate receptor/gamma-adaptin vesicles at the trans-Golgi network. J. Biol. Chem..

[57] Pols M.S., van Meel E., Oorschot V., ten Brink C., Fukuda M., Swetha M.G., Mayor S., Klumperman J. (2013). hVps41 and VAMP7 function in direct TGN to late endosome transport of lysosomal membrane proteins. Nat. Commun..

[58] Pols M.S., Klumperman J. (2009). Trafficking and function of the tetraspanin CD63. Exp. Cell Res..

[59] Varki A., Kornfeld S., Varki A., Cummings R.D., Esko J.D., Freeze H.H., Stanley P., Bertozzi C.R., Hart G.W., Etzler M.E. (2009). P-type lectins. Essentials of Glycobiology.

[60] Kim J.-J.P., Olson L.J., Dahms N.M. (2009). Carbohydrate recognition by the mannose-6-phosphate receptors. Curr. Opin. Struct. Biol..

[61] Pohl S., Marschner K., Storch S., Braulke T. (2009). Glycosylation- and phosphorylation-dependent intracellular transport of lysosomal hydrolases. Biol. Chem..

[62] Kornfeld S., Sly W.S., Schriver C.R., Baudet A.L., Sly W.S., Valle D. (2000). I-cell disease and pseudo-Hurler polydystrophy: Disorders of lysosomal enzyme phosphorylation and localization. The Metabolic and Molecular Bases of Inherited Disease.

[63] Leroy J.G., Cathey S., Friez M.J., Pagon R.A., Adam M.P., Ardinger H.H., Wallace S.E., Amemiya A., Bean L.J., Bird T.D., Ledbetter N., Mefford H.C., Smith R.J. (1993). Mucolipidosis II. GeneReviews^®^.

[64] Waheed A., Pohlmann R., Hasilik A., von Figura K., van Elsen A., Leroy J.G. (1982). Deficiency of UDP-*N*-acetylglucosamine: Lysosomal enzyme *N*-acetylglucosamine-1-phosphotransferase in organs of I-cell patients. Biochem. Biophys. Res. Commun..

[65] Owada M., Neufeld E.F. (1982). Is there a mechanism for introducing acid hydrolases into liver lysosomes that is independent of mannose 6-phosphate recognition? Evidence from I-cell disease. Biochem. Biophys. Res. Commun..

[66] Little L., Alcouloumre M., Drotar A.M., Herman S., Robertson R., Yeh R.Y., Miller A.L. (1987). Properties of *N*-acetylglucosamine 1-phosphotransferase from human lymphoblasts. Biochem. J..

[67] Glickman J.N., Kornfeld S. (1993). Mannose 6-phosphate-independent targeting of lysosomal enzymes in I-cell disease B lymphoblasts. J. Cell Biol..

[68] Boonen M., van Meel E., Oorschot V., Klumperman J., Kornfeld S. (2011). Vacuolization of mucolipidosis type II mouse exocrine gland cells represents accumulation of autolysosomes. Mol. Biol. Cell.

[69] van Meel E., Boonen M., Zhao H., Oorschot V., Ross F.P., Kornfeld S., Klumperman J. (2011). Disruption of the Man-6-P targeting pathway in mice impairs osteoclast secretory lysosome biogenesis. Traffic (Cph. Den.).

[70] Kollmann K., Pestka J.M., Kühn S.C., Schöne E., Schweizer M., Karkmann K., Otomo T., Catala-Lehnen P., Failla A.V., Marshall R.P. (2013). Decreased bone formation and increased osteoclastogenesis cause bone loss in mucolipidosis II. EMBO Mol. Med..

[71] Kollmann K., Damme M., Markmann S., Morelle W., Schweizer M., Hermans-Borgmeyer I., Röchert A.K., Pohl S., Lübke T., Michalski J.-C. (2012). Lysosomal dysfunction causes neurodegeneration in mucolipidosis II “knock-in” mice. Brain J. Neurol..

[72] Idol R.A., Wozniak D.F., Fujiwara H., Yuede C.M., Ory D.S., Kornfeld S., Vogel P. (2014). Neurologic abnormalities in mouse models of the lysosomal storage disorders mucolipidosis II and mucolipidosis III γ. PLoS ONE.

[73] Dittmer F., Ulbrich E.J., Hafner A., Schmahl W., Meister T., Pohlmann R., von Figura K. (1999). Alternative mechanisms for trafficking of lysosomal enzymes in mannose 6-phosphate receptor-deficient mice are cell type-specific. J. Cell Sci..

[74] Hogue D.L., Nash C., Ling V., Hobman T.C. (2002). Lysosome-associated protein transmembrane 4 α (LAPTM4 α) requires two tandemly arranged tyrosine-based signals for sorting to lysosomes. Biochem. J..

[75] Dell’Angelica E.C., Shotelersuk V., Aguilar R.C., Gahl W.A., Bonifacino J.S. (1999). Altered trafficking of lysosomal proteins in Hermansky-Pudlak syndrome due to mutations in the β 3A subunit of the AP-3 adaptor. Mol. Cell.

[76] Rous B.A., Reaves B.J., Ihrke G., Briggs J.A.G., Gray S.R., Stephens D.J., Banting G., Luzio J.P. (2002). Role of adaptor complex AP-3 in targeting wild-type and mutated CD63 to lysosomes. Mol. Biol. Cell.

[77] Hirst J., Bright N.A., Rous B., Robinson M.S. (1999). Characterization of a fourth adaptor-related protein complex. Mol. Biol. Cell.

[78] Janvier K., Bonifacino J.S. (2005). Role of the endocytic machinery in the sorting of lysosome-associated membrane proteins. Mol. Biol. Cell.

[79] Harter C., Mellman I. (1992). Transport of the lysosomal membrane glycoprotein lgp120 (lgp-A) to lysosomes does not require appearance on the plasma membrane. J. Cell Biol..

[80] Rohrer J., Schweizer A., Russell D., Kornfeld S. (1996). The targeting of Lamp1 to lysosomes is dependent on the spacing of its cytoplasmic tail tyrosine sorting motif relative to the membrane. J. Cell Biol..

[81] Obermüller S., Kiecke C., von Figura K., Höning S. (2002). The tyrosine motifs of Lamp 1 and LAP determine their direct and indirect targetting to lysosomes. J. Cell Sci..

[82] Peden A.A., Oorschot V., Hesser B.A., Austin C.D., Scheller R.H., Klumperman J. (2004). Localization of the AP-3 adaptor complex defines a novel endosomal exit site for lysosomal membrane proteins. J. Cell Biol..

[83] Chapuy B., Tikkanen R., Mühlhausen C., Wenzel D., von Figura K., Höning S. (2008). AP-1 and AP-3 mediate sorting of melanosomal and lysosomal membrane proteins into distinct post-Golgi trafficking pathways. Traffic (Cph. Den.).

[84] Aguilar R.C., Boehm M., Gorshkova I., Crouch R.J., Tomita K., Saito T., Ohno H., Bonifacino J.S. (2001). Signal-binding specificity of the mu4 subunit of the adaptor protein complex AP-4. J. Biol. Chem..

[85] Lefrancois S., Zeng J., Hassan A.J., Canuel M., Morales C.R. (2003). The lysosomal trafficking of sphingolipid activator proteins (SAPs) is mediated by sortilin. EMBO J..

[86] Canuel M., Lefrancois S., Zeng J., Morales C.R. (2008). AP-1 and retromer play opposite roles in the trafficking of sortilin between the Golgi apparatus and the lysosomes. Biochem. Biophys. Res. Commun..

[87] Nielsen M.S., Madsen P., Christensen E.I., Nykjaer A., Gliemann J., Kasper D., Pohlmann R., Petersen C.M. (2001). The sortilin cytoplasmic tail conveys Golgi-endosome transport and binds the VHS domain of the GGA2 sorting protein. EMBO J..

[88] Takatsu H., Katoh Y., Shiba Y., Nakayama K. (2001). Golgi-localizing, gamma-adaptin ear homology domain, ADP-ribosylation factor-binding (GGA) proteins interact with acidic dileucine sequences within the cytoplasmic domains of sorting receptors through their Vps27p/Hrs/STAM (VHS) domains. J. Biol. Chem..

[89] Hassan A.J., Zeng J., Ni X., Morales C.R. (2004). The trafficking of prosaposin (SGP-1) and GM2AP to the lysosomes of TM4 Sertoli cells is mediated by sortilin and monomeric adaptor proteins. Mol. Reprod. Dev..

[90] Le Borgne R., Alconada A., Bauer U., Hoflack B. (1998). The mammalian AP-3 adaptor-like complex mediates the intracellular transport of lysosomal membrane glycoproteins. J. Biol. Chem..

[91] Höning S., Sandoval I.V., von Figura K. (1998). A di-leucine-based motif in the cytoplasmic tail of LIMP-II and tyrosinase mediates selective binding of AP-3. EMBO J..

[92] Fujita H., Saeki M., Yasunaga K., Ueda T., Imoto T., Himeno M. (1999). In vitro binding study of adaptor protein complex (AP-1) to lysosomal targeting motif (LI-motif). Biochem. Biophys. Res. Commun..

[93] Tabuchi N., Akasaki K., Tsuji H. (2000). Two acidic amino acid residues, Asp(470) and Glu(471), contained in the carboxyl cytoplasmic tail of a major lysosomal membrane protein, LGP85/LIMP II, are important for its accumulation in secondary lysosomes. Biochem. Biophys. Res. Commun..

[94] Tabuchi N., Akasaki K., Tsuji H. (2002). Ile (476), a constituent of di-leucine-based motif of a major lysosomal membrane protein, LGP85/LIMP II, is important for its proper distribution in late endosomes and lysosomes. Biochem. Biophys. Res. Commun..

[95] Janvier K., Kato Y., Boehm M., Rose J.R., Martina J.A., Kim B.-Y., Venkatesan S., Bonifacino J.S. (2003). Recognition of dileucine-based sorting signals from HIV-1 Nef and LIMP-II by the AP-1 γ-sigma1 and AP-3 δ/σ3 hemicomplexes. J. Cell Biol..

[96] Chen W.J., Goldstein J.L., Brown M.S. (1990). NPXY, a sequence often found in cytoplasmic tails, is required for coated pit-mediated internalization of the low density lipoprotein receptor. J. Biol. Chem..

[97] Garcia C.K., Wilund K., Arca M., Zuliani G., Fellin R., Maioli M., Calandra S., Bertolini S., Cossu F., Grishin N. (2001). Autosomal recessive hypercholesterolemia caused by mutations in a putative LDL receptor adaptor protein. Science.

[98] Eden E.R., Patel D.D., Sun X.-M., Burden J.J., Themis M., Edwards M., Lee P., Neuwirth C., Naoumova R.P., Soutar A.K. (2002). Restoration of LDL receptor function in cells from patients with autosomal recessive hypercholesterolemia by retroviral expression of ARH1. J. Clin. Investig..

[99] Boll W., Rapoport I., Brunner C., Modis Y., Prehn S., Kirchhausen T. (2002). The mu2 subunit of the clathrin adaptor AP-2 binds to FDNPVY and YppØ sorting signals at distinct sites. Traffic (Cph. Den.).

[100] Li Y., Marzolo M.P., van Kerkhof P., Strous G.J., Bu G. (2000). The YXXL motif, but not the two NPXY motifs, serves as the dominant endocytosis signal for low density lipoprotein receptor-related protein. J. Biol. Chem..

[101] Guttman M., Betts G.N., Barnes H., Ghassemian M., van der Geer P., Komives E.A. (2009). Interactions of the NPXY microdomains of the low density lipoprotein receptor-related protein 1. Proteomics.

[102] Oleinikov A.V., Zhao J., Makker S.P. (2000). Cytosolic adaptor protein Dab2 is an intracellular ligand of endocytic receptor gp600/megalin. Biochem. J..

[103] Takeda T., Yamazaki H., Farquhar M.G. (2003). Identification of an apical sorting determinant in the cytoplasmic tail of megalin. Am. J. Physiol. Cell Physiol..

[104] Nagai M., Meerloo T., Takeda T., Farquhar M.G. (2003). The adaptor protein ARH escorts megalin to and through endosomes. Mol. Biol. Cell.

[105] Boonen M., Staudt C., Gilis F., Oorschot V., Klumperman J., Jadot M. (2016). Cathepsin D and its newly identified transport receptor SEZ6L2 can modulate neurite outgrowth. J. Cell Sci..

[106] East L., Isacke C.M. (2002). The mannose receptor family. Biochim. Biophys. Acta.

[107] Kruskal B.A., Sastry K., Warner A.B., Mathieu C.E., Ezekowitz R.A. (1992). Phagocytic chimeric receptors require both transmembrane and cytoplasmic domains from the mannose receptor. J. Exp. Med..

[108] Beaujouin M., Prébois C., Derocq D., Laurent-Matha V., Masson O., Pattingre S., Coopman P., Bettache N., Grossfield J., Hollingsworth R.E. (2010). Pro-cathepsin D interacts with the extracellular domain of the β chain of LRP1 and promotes LRP1-dependent fibroblast outgrowth. J. Cell Sci..

[109] Zunke F., Andresen L., Wesseler S., Groth J., Arnold P., Rothaug M., Mazzulli J.R., Krainc D., Blanz J., Saftig P. (2016). Characterization of the complex formed by β-glucocerebrosidase and the lysosomal integral membrane protein type-2. Proc. Natl. Acad. Sci. USA.

[110] Westergaard U.B., Sørensen E.S., Hermey G., Nielsen M.S., Nykjaer A., Kirkegaard K., Jacobsen C., Gliemann J., Madsen P., Petersen C.M. (2004). Functional organization of the sortilin Vps10p domain. J. Biol. Chem..

[111] van Meel E., Klumperman J. (2008). Imaging and imagination: Understanding the endo-lysosomal system. Histochem. Cell Biol..

[112] Coutinho M.F., Prata M.J., Alves S. (2012). Mannose-6-phosphate pathway: A review on its role in lysosomal function and dysfunction. Mol. Genet. Metab..

[113] Reczek D., Schwake M., Schröder J., Hughes H., Blanz J., Jin X., Brondyk W., van Patten S., Edmunds T., Saftig P. (2007). LIMP-2 is a receptor for lysosomal mannose-6-phosphate-independent targeting of β-glucocerebrosidase. Cell.

[114] Kuronita T., Eskelinen E.-L., Fujita H., Saftig P., Himeno M., Tanaka Y. (2002). A role for the lysosomal membrane protein LGP85 in the biogenesis and maintenance of endosomal and lysosomal morphology. J. Cell Sci..

[115] Rothaug M., Zunke F., Mazzulli J.R., Schweizer M., Altmeppen H., Lüllmann-Rauch R., Kallemeijn W.W., Gaspar P., Aerts J.M., Glatzel M. (2014). LIMP-2 expression is critical for β-glucocerebrosidase activity and α-synuclein clearance. Proc. Natl. Acad. Sci. USA.

[116] Ni X., Morales C.R. (2006). The lysosomal trafficking of acid sphingomyelinase is mediated by sortilin and mannose 6-phosphate receptor. Traffic (Cph. Den.).

[117] Canuel M., Korkidakis A., Konnyu K., Morales C.R. (2008). Sortilin mediates the lysosomal targeting of cathepsins D and H. Biochem. Biophys. Res. Commun..

[118] Zeng J., Racicott J., Morales C.R. (2009). The inactivation of the sortilin gene leads to a partial disruption of prosaposin trafficking to the lysosomes. Exp. Cell Res..

[119] Canuel M., Libin Y., Morales C.R. (2009). The interactomics of sortilin: An ancient lysosomal receptor evolving new functions. Histol. Histopathol..

[120] Wähe A., Kasmapour B., Schmaderer C., Liebl D., Sandhoff K., Nykjaer A., Griffiths G., Gutierrez M.G. (2010). Golgi-to-phagosome transport of acid sphingomyelinase and prosaposin is mediated by sortilin. J. Cell Sci..

[121] Markmann S., Thelen M., Cornils K., Schweizer M., Brocke-Ahmadinejad N., Willnow T., Heeren J., Gieselmann V., Braulke T., Kollmann K. (2015). Lrp1/LDL receptor play critical roles in mannose 6-phosphate-independent lysosomal enzyme targeting. Traffic (Cph. Den.).

[122] Christensen E.I., Zhou Q., Sørensen S.S., Rasmussen A.K., Jacobsen C., Feldt-Rasmussen U., Nielsen R. (2007). Distribution of α-galactosidase A in normal human kidney and renal accumulation and distribution of recombinant alpha-galactosidase A in Fabry mice. J. Am. Soc. Nephrol. JASN.

[123] Prabakaran T., Nielsen R., Larsen J.V., Sørensen S.S., Feldt-Rasmussen U., Saleem M.A., Petersen C.M., Verroust P.J., Christensen E.I. (2011). Receptor-mediated endocytosis of α-galactosidase A in human podocytes in Fabry disease. PLoS ONE.

[124] Nielsen R., Courtoy P.J., Jacobsen C., Dom G., Lima W.R., Jadot M., Willnow T.E., Devuyst O., Christensen E.I. (2007). Endocytosis provides a major alternative pathway for lysosomal biogenesis in kidney proximal tubular cells. Proc. Natl. Acad. Sci. USA.

[125] Derocq D., Prébois C., Beaujouin M., Laurent-Matha V., Pattingre S., Smith G.K., Liaudet-Coopman E. (2012). Cathepsin D is partly endocytosed by the LRP1 receptor and inhibits LRP1-regulated intramembrane proteolysis. Oncogene.

[126] Liaudet-Coopman E., Beaujouin M., Derocq D., Garcia M., Glondu-Lassis M., Laurent-Matha V., Prébois C., Rochefort H., Vignon F. (2006). Cathepsin D: Newly discovered functions of a long-standing aspartic protease in cancer and apoptosis. Cancer Lett..

[127] Laurent-Matha V., Farnoud M.R., Lucas A., Rougeot C., Garcia M., Rochefort H. (1998). Endocytosis of pro-cathepsin D into breast cancer cells is mostly independent of mannose-6-phosphate receptors. J. Cell Sci..

[128] Stützer I., Selevsek N., Esterházy D., Schmidt A., Aebersold R., Stoffel M. (2013). Systematic proteomic analysis identifies β-site amyloid precursor protein cleaving enzyme 2 and 1 (BACE2 and BACE1) substrates in pancreatic β-cells. J. Biol. Chem..

[129] Kuhn P.-H., Koroniak K., Hogl S., Colombo A., Zeitschel U., Willem M., Volbracht C., Schepers U., Imhof A., Hoffmeister A. (2012). Secretome protein enrichment identifies physiological BACE1 protease substrates in neurons. EMBO J..

[130] Miyazaki T., Hashimoto K., Uda A., Sakagami H., Nakamura Y., Saito S., Nishi M., Kume H., Tohgo A., Kaneko I. (2006). Disturbance of cerebellar synaptic maturation in mutant mice lacking BSRPs, a novel brain-specific receptor-like protein family. FEBS Lett..

[131] Gunnersen J.M., Kim M.H., Fuller S.J., de Silva M., Britto J.M., Hammond V.E., Davies P.J., Petrou S., Faber E.S.L., Sah P. (2007). Sez-6 proteins affect dendritic arborization patterns and excitability of cortical pyramidal neurons. Neuron.

[132] Achord D.T., Brot F.E., Bell C.E., Sly W.S. (1978). Human beta-glucuronidase: In vivo clearance and in vitro uptake by a glycoprotein recognition system on reticuloendothelial cells. Cell.

[133] Hubbard A.L., Wilson G., Ashwell G., Stukenbrok H. (1979). An electron microscope autoradiographic study of the carbohydrate recognition systems in rat liver. I. Distribution of 125I-ligands among the liver cell types. J. Cell Biol..

[134] Maynard Y., Baenziger J.U. (1981). Oligosaccharide specific endocytosis by isolated rat hepatic reticuloendothelial cells. J. Biol. Chem..

[135] Lennartz M.R., Wileman T.E., Stahl P.D. (1987). Isolation and characterization of a mannose-specific endocytosis receptor from rabbit alveolar macrophages. Biochem. J..

[136] Taylor M.E., Conary J.T., Lennartz M.R., Stahl P.D., Drickamer K. (1990). Primary structure of the mannose receptor contains multiple motifs resembling carbohydrate-recognition domains. J. Biol. Chem..

[137] Lee S.J., Evers S., Roeder D., Parlow A.F., Risteli J., Risteli L., Lee Y.C., Feizi T., Langen H., Nussenzweig M.C. (2002). Mannose receptor-mediated regulation of serum glycoprotein homeostasis. Science.

[138] Elvevold K., Simon-Santamaria J., Hasvold H., McCourt P., Smedsrød B., Sørensen K.K. (2008). Liver sinusoidal endothelial cells depend on mannose receptor-mediated recruitment of lysosomal enzymes for normal degradation capacity. Hepatology.

[139] Sleat D.E., Della Valle M.C., Zheng H., Moore D.F., Lobel P. (2008). The mannose 6-phosphate glycoprotein proteome. J. Proteome Res..

[140] Puissant E., Gilis F., Dogné S., Flamion B., Jadot M., Boonen M. (2014). Subcellular trafficking and activity of Hyal-1 and its processed forms in murine macrophages. Traffic (Cph. Den.).

[141] Boonen M., Puissant E., Gilis F., Flamion B., Jadot M. (2014). Mouse liver lysosomes contain enzymatically active processed forms of Hyal-1. Biochem. Biophys. Res. Commun..

[142] Furbish F.S., Steer C.J., Krett N.L., Barranger J.A. (1981). Uptake and distribution of placental glucocerebrosidase in rat hepatic cells and effects of sequential deglycosylation. Biochim. Biophys. Acta.

[143] Brady R.O., Murray G.J., Barton N.W. (1994). Modifying exogenous glucocerebrosidase for effective replacement therapy in Gaucher disease. J. Inherit. Metab. Dis..

[144] Puissant E., Boonen M. (2016). Monocytes/macrophages upregulate the hyaluronidase HYAL1 and adapt its subcellular trafficking to promote extracellular residency upon differentiation into osteoclasts. PLoS ONE.

[145] Kowalewski B., Lübke T., Kollmann K., Braulke T., Reinheckel T., Dierks T., Damme M. (2014). Molecular characterization of arylsulfatase G: Expression, processing, glycosylation, transport, and activity. J. Biol. Chem..

[146] Tsuji A., Omura K., Suzuki Y. (1988). Intracellular transport of acid α-glucosidase in human fibroblasts: Evidence for involvement of phosphomannosyl receptor-independent system. J. Biochem. (Tokyo).

[147] Díaz E., Pfeffer S.R. (1998). TIP47: A cargo selection device for mannose 6-phosphate receptor trafficking. Cell.

[148] Carroll K.S., Hanna J., Simon I., Krise J., Barbero P., Pfeffer S.R. (2001). Role of Rab9 GTPase in facilitating receptor recruitment by TIP47. Science.

[149] Puertollano R. (2014). mTOR and lysosome regulation. F1000prime Rep..

[150] Efeyan A., Zoncu R., Sabatini D.M. (2012). Amino acids and mTORC1: From lysosomes to disease. Trends Mol. Med..

[151] Zoncu R., Bar-Peled L., Efeyan A., Wang S., Sancak Y., Sabatini D.M. (2011). mTORC1 senses lysosomal amino acids through an inside-out mechanism that requires the vacuolar H(+)-ATPase. Science.

[152] Stransky L.A., Forgac M. (2015). Amino acid availability modulates vacuolar H+-ATPase assembly. J. Biol. Chem..

[153] Hirst J., Borner G.H.H., Edgar J., Hein M.Y., Mann M., Buchholz F., Antrobus R., Robinson M.S. (2013). Interaction between AP-5 and the hereditary spastic paraplegia proteins SPG11 and SPG15. Mol. Biol. Cell.

[154] Chang J., Lee S., Blackstone C. (2014). Spastic paraplegia proteins spastizin and spatacsin mediate autophagic lysosome reformation. J. Clin. Investig..

[155] Renvoisé B., Chang J., Singh R., Yonekawa S., FitzGibbon E.J., Mankodi A., Vanderver A., Schindler A., Toro C., Gahl W.A. (2014). Lysosomal abnormalities in hereditary spastic paraplegia types SPG15 and SPG11. Ann. Clin. Transl. Neurol..

[156] Giuliani F., Grieve A., Rabouille C. (2011). Unconventional secretion: A stress on GRASP. Curr. Opin. Cell Biol..

[157] Nickel W., Rabouille C. (2009). Mechanisms of regulated unconventional protein secretion. Nat. Rev. Mol. Cell Biol..

[158] Deretic V., Jiang S., Dupont N. (2012). Autophagy intersections with conventional and unconventional secretion in tissue development, remodeling and inflammation. Trends Cell Biol..

[159] Rabouille C., Linstedt A.D. (2016). GRASP: A multitasking tether. Front. Cell Dev. Biol..

[160] Young A.R.J., Chan E.Y.W., Hu X.W., Köchl R., Crawshaw S.G., High S., Hailey D.W., Lippincott-Schwartz J., Tooze S.A. (2006). Starvation and ULK1-dependent cycling of mammalian Atg9 between the TGN and endosomes. J. Cell Sci..

[161] Staudt C., Gilis F., Boonen M., Jadot M. (2016). Molecular determinants that mediate the sorting of human ATG9A from the endoplasmic reticulum. Biochim. Biophys. Acta.

[162] Tveit H., Akslen L.K.A., Fagereng G.L., Tranulis M.A., Prydz K. (2009). A secretory Golgi bypass route to the apical surface domain of epithelial MDCK cells. Traffic (Cph. Den.).

[163] Syres K., Harrison F., Tadlock M., Jester J.V., Simpson J., Roy S., Salomon D.R., Cherqui S. (2009). Successful treatment of the murine model of cystinosis using bone marrow cell transplantation. Blood.

[164] Rocca C.J., Kreymerman A., Ur S.N., Frizzi K.E., Naphade S., Lau A., Tran T., Calcutt N.A., Goldberg J.L., Cherqui S. (2015). Treatment of inherited eye defects by systemic hematopoietic stem cell transplantation. Investig. Ophthalmol. Vis. Sci..

[165] Gaide Chevronnay H.P., Janssens V., van der Smissen P., Rocca C.J., Liao X.H., Refetoff S., Pierreux C.E., Cherqui S., Courtoy P.J. (2016). Hematopoietic stem cells transplantation can normalize thyroid function in a cystinosis mouse model. Endocrinology.

[166] Naphade S., Sharma J., Gaide Chevronnay H.P., Shook M.A., Yeagy B.A., Rocca C.J., Ur S.N., Lau A.J., Courtoy P.J., Cherqui S. (2015). Brief reports: Lysosomal cross-correction by hematopoietic stem cell-derived macrophages via tunneling nanotubes. Stem Cells.

[167] Rustom A., Saffrich R., Markovic I., Walther P., Gerdes H.-H. (2004). Nanotubular highways for intercellular organelle transport. Science.

[168] Rustom A. (2016). The missing link: Does tunnelling nanotube-based supercellularity provide a new understanding of chronic and lifestyle diseases?. Open Biol..

[169] Cocucci E., Meldolesi J. (2015). Ectosomes and exosomes: Shedding the confusion between extracellular vesicles. Trends Cell Biol..

[170] Iglesias D.M., El-Kares R., Taranta A., Bellomo F., Emma F., Besouw M., Levtchenko E., Toelen J., van den Heuvel L., Chu L. (2012). Stem cell microvesicles transfer cystinosin to human cystinotic cells and reduce cystine accumulation in vitro. PLoS ONE.

[171] Keerthikumar S., Chisanga D., Ariyaratne D., Al Saffar H., Anand S., Zhao K., Samuel M., Pathan M., Jois M., Chilamkurti N. (2016). ExoCarta: A web-based compendium of exosomal cargo. J. Mol. Biol..

[172] Qian M., Sleat D.E., Zheng H., Moore D., Lobel P. (2008). Proteomics analysis of serum from mutant mice reveals lysosomal proteins selectively transported by each of the two mannose 6-phosphate receptors. Mol. Cell. Proteom. MCP.

[173] Matzner U., von Figura K., Pohlmann R. (1992). Expression of the two mannose 6-phosphate receptors is spatially and temporally different during mouse embryogenesis. Development (Camb.).

[174] Waguri S., Kohmura M., Kanamori S., Watanabe T., Ohsawa Y., Koike M., Tomiyama Y., Wakasugi M., Kominami E., Uchiyama Y. (2001). Different distribution patterns of the two mannose 6-phosphate receptors in rat liver. J. Histochem. Cytochem..

[175] Konishi Y., Fushimi S., Shirabe T. (2005). Immunohistochemical distribution of cation-dependent mannose 6-phosphate receptors in the mouse central nervous system: Comparison with that of cation-independent mannose 6-phophate receptors. Neurosci. Lett..

[176] Uhlén M., Fagerberg L., Hallström B.M., Lindskog C., Oksvold P., Mardinoglu A., Sivertsson Å., Kampf C., Sjöstedt E., Asplund A. (2015). Proteomics. Tissue-based map of the human proteome. Science.

